# Endophytic fungus *Phomopsis* sp. as a source of 3-nitropropionic acid with larvicidal activity against *Aedes aegypti* (Linnaeus 1762, Diptera: Culicidae)

**DOI:** 10.1590/0037-8682-0018-2022

**Published:** 2022-10-24

**Authors:** Armando da Costa Garcia, Orivaldo Teixeira de Menezes, Lissa Apolonia Mariano, Letícia Corrêa Santiago, Ângela Regina Araújo, Julia Dietsche Monfardini, Rejane de Castro Simões, André Correa de Oliveira, Rosemary Aparecida Roque, Wanderli Pedro Tadei, Helder Lopes Teles, Camila Martins de Oliveira

**Affiliations:** 1 Universidade Federal do Amazonas, Instituto de Ciências Exatas e Tecnologia, Itacoatiara, AM, Brasil.; 2 Universidade Estadual Paulista Júlio de Mesquita Filho, Araraquara, SP, Brasil.; 3 Instituto Nacional de Pesquisas da Amazônia, Manaus, AM, Brasil.; 4 Universidade Federal de Rondonópolis, Instituto de Ciências Exatas e Naturais, Rondonópolis, MT, Brasil.

**Keywords:** Biomonitored chemical, Secondary metabolite, Dengue

## Abstract

**Background:**

*Aedes aegypti* is the primary vector of viruses, such as Zika, chikungunya, yellow fever, and dengue. In this context, a biomonitored chemical study was conducted to evaluate the activity of the crude extract of the endophytic fungus *Phomopsis* sp. against the larvae of *Aedes aegypti*.

**Methods:**

Crude extract, fractions, and isolated substances were evaluated in in-vitro assays against third-stage larvae of *Aedes aegypti*.

**Results:**

We isolated 3-nitropropionic acid with an LC_50_ of 15.172 ppm and LC_90_ of 18.178 ppm after 24 hours of larval exposure.

**Conclusions:**

The results indicated that 3-nitropropionic acid exerted larvicidal activity.

The mosquito *Aedes aegypti* (Linnaeus 1762; Diptera: Culicidae) is responsible for the transmission of the Zika, chikungunya, yellow fever, and dengue viruses, all of which are a cause for concern in health sectors around the world^1^. In Brazil, the dengue virus is considered hyperendemic with the circulation of all four serotypes (DENV-1 to DENV-4)^2^. According to the epidemiological bulletin of the Brazil Ministry of Health^3^, in the first 4 months of 2022 alone there was a 151.4% increase in registered dengue cases compared with the same period of the previous year.

Synthetic insecticides are the main means of controlling the vectors of these diseases; however, the continuous use of these products has triggered resistance in mosquito populations, as well as affecting non-target organisms and contaminating the environment^1^. Therefore, it is critical to find new ways to combat transmitting agents, such as natural fungal products, which have shown great potential.

Fungi of the genus *Phomopsis* are widely distributed and have been identified as a rich source of new compounds with several biological activities^4^. Accordingly, 3-nitropropionic acid (3-NPA) has been isolated from fungal strains of the genus *Phomopsis* ^4^. This acid is known as a neurotoxic substance in mammals and is frequently used as an inducer of Huntington's disease symptoms for evaluation in in-vivo animal models^5^. 

The current literature provides few studies involving endophytic fungi with activity against the larvae of *Aedes aegypti*. Therefore, the aim of this study was to conduct a biomonitored chemical analysis and obtain a pure substance that could be used as an alternative control for the transmission agent of Zika, chikungunya, yellow fever, and dengue viruses.

The endophytic fungus *Phomopsis* sp. was isolated from the plant species *Passovia stelis* (L.) Kuijt (Lorantahaceae) following the method of Oliveira et al.^6^ The plant was collected in February 2012 on the campus of the Federal University of Amazonas (UFAM; 3°08'57"S, 58°26'38"W) in the city of Itacoatiara, Amazonas, Brazil. The species was identified by Dr. Welma Sousa Silva Carneiro of the UFAM Institute of Exact Sciences and Technology. A voucher specimen (No. 244061) was deposited in the herbarium of the National Research Institute of the Amazon (INPA; Manaus, Brazil).

The fungus was identified according to the morphological characteristics of the culture and by comparing the genetic sequencing of rDNA extracted from the mycelia. The extraction was performed according to the protocol described by Ribeiro et al.^7^ The internal transcribed spacer region was amplified and bidirectionally sequenced using Sanger sequencing. The generated sequences were compared with reference data available in the GenBank database, indicating 98% similarity with the genus *Phomopsis* sp.^7^

To obtain the crude extract, *Phomopsis* sp. was grown in 72 500 mL Erlenmeyer flasks, each containing 250 mL of the aqueous culture medium, dextrose potato broth, previously autoclaved at 121°C for 15 minutes. The endophyte was grown in Petri dishes with a potato dextrose agar medium for 5 days in a biochemical oxygen demand incubator. After sterilization, five pieces (1 cm^2^) of the endophyte were inoculated. The 72 inoculated vials were incubated at 25°C for 20 days. After this period, the broth was separated from the mycelium by filtering and subjected to liquid-liquid partition with ethyl acetate (EtOAc) (3 × 125 mL). The organic phase was concentrated under reduced pressure, resulting in 2.94 g of crude extract. After confirmation of larvicidal activity, the crude extract was fractionated in a silica normal phase cartridge using an increasing polarity gradient (dichloromethane, EtOAc, and methane [MeOH]), resulting in 19 fractions. Fractions with similar patterns in the comparative thin layer chromatography were collected from F1 to F8 and subjected to evaluation of selective larvicidal activity, with F4 being the most active. This was fractionated by column chromatography packaged with octyldecylsilane and eluted with MeOH:H_2_O 9:1 (v/v), resulting in 10 subfractions (F4-1 to F4-10). The F4-1 subfraction showed better purity and, through spectroscopic analysis and comparison with data available in the literature, it was possible to identify 3-NPA, which was subjected to a bioassay against the larvae of *Aedes aegypti*.

The larvicidal bioassays were conducted according to the protocol recommended by the World Health Organization^8^. The bioassays were conducted at a temperature of 26 ± 2°C and relative humidity of 85%. The selective bioassay was conducted with extracts and fractions at concentrations of 100 and 500 ppm, previously diluted in dimethyl sulfoxide (DMSO), also used as the negative control, at 1% (v/v). The assay was conducted in triplicate in 50 mL cups, each of which contained 10 third-stage larvae of *Aedes aegypti* and 40 µL of larval food. Mortality was determined at 24, 48, and 72 hours after the start of the bioassay. 

Evaluations of 3-NPA were done at concentrations of 20, 18, 16, 14, and 12 ppm, in triplicate. The bioassay was performed in 50 mL cups containing 9.8 mL of well water, 100 µL of larval food, 10 third-stage larvae of *Aedes aegypti*, and 100 µL of solutions of 3-NPA previously diluted in 1% DMSO (v/v). The negative control was assembled of 9.8 mL of well water, 10 third-stage larvae of *Aedes aegypti*, 100 µL of larval food, and 100 µL DMSO, corresponding to a concentration of 1% (v/v). The positive control was assembled using *Bacillus thuringiensis israelensis* (Bti) at 1 ppm. Larval mortality readings were performed 24 and 48 hours after contact with 3-NPA. Larvae that did not respond to the artificial stimuli were considered dead.

The Chi-square, slope ± SE, and R^2^ tests were used to indicate the non-significance of the lethal concentration values, growth up the curve of mortality, and the linear correlation between concentrations and mortality, calculated using Poloplus software version 1.0. (LeOra Software). The data were analyzed by one-way analysis of variance (ANOVA) and Tukey’s test (*p*<0.05) using GraphPad Prism software (version 6.0; San Diego, CA, USA) and were expressed as mean (%) ± standard deviation.

The extract caused the death of 18 of 30 larvae at a concentration of 100 ppm, representing 60% mortality after 72 hours of exposure. At 500 ppm, 100% mortality was observed within 24 hours. Fraction 4 showed 100% larval mortality at the two tested concentrations (100 and 500 ppm) in the first 24 hours. No larval death was observed in the control group. 

The biomonitored investigation of the crude extract of *Phomopsis* sp. allowed the isolation of 3-NPA (Figure 1). Its structure was elucidated based on ^1^H NMR spectroscopic data and by comparison with data obtained by Flores et al.^4^ (Table 1). 

**FIGURE 1:**
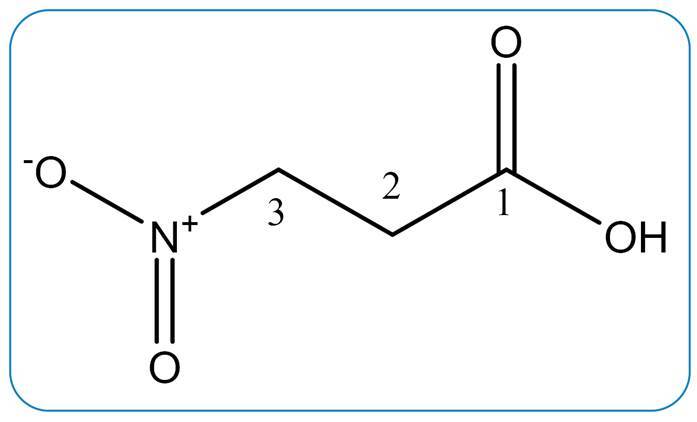
Chemical structure of 3-nitropropionic acid.

**TABLE 1:** ^1^H NMR data of 3-nitropropionic acid obtained at 300 MHz.

Position	Experimental	(FLORES et al., 2013)
	δ_H_ (multiplicity, position, coupling)*	δ_H_ (multiplicity, position, coupling)*
2	2.87 (t, *J* = 5.9 Hz)	2.84 (t, *J* = 6.3 Hz)
3	4.69 (t, *J* = 5.9 Hz)	4.64 (t, *J* = 6.3 Hz)

**δ**_H_ **:** Chemical displacement; *Experiment conducted in DMSO-d_6_.

The ^1^H NMR spectrum showed two triplet signals at *δ*_H_ 2.87 (2H, *t*, *J* = 5.9 Hz, H-2) and 4.69 (2H, *t*, *J* = 5.9 Hz, H-3), attributed to the coupling ^3^ *J* of the vicinal methylene hydrogens H-2 and H-3. The chemical displacement at *δ*_H2_ 2.87 is characteristic of carboxylic α-hydrogens (-COOH) under greater deprotection caused by the nitro group (-NO_2_) in position b at H-2. Similarly, the *δ*_H3_ signal 4.69 suffers the greatest influence of deprotection due to the position a of the nitro group (-NO_2_). 

The results of the larvicidal bioassays with 3-NPA (Table 2) indicated an LC_50_ of 15.172 ppm and an LC_90_ of 18.178 ppm against third-stage larvae of *Aedes aegypti*. No larval mortality was observed in the negative control, indicating that the observed larvicidal activity was related to the action of 3-NPA.

**TABLE 2:** Percentages of mortality of *A. aegypti* larvae and lethal doses of 3-nitropropionic acid after 24 h of larval exposure.

Concentrations	Mortality	LC_50_ (ppm)	LC_90_ (ppm)				
(ppm)	(%)	(LCL-UCL)	(LCL-UCL)	χ^2^ (d.f.)	Slope ± SE	R^2^	*p*-value*
12	4 ± 1^e^	15.172 (14.440 - 15.873)	18.178 (17.192 - 19.932)	5.5 (3)	16.3214 ± 1.195	0.9814	<0.001
14	32.2 ± 2^d^						
16	64.4 ± 2^c^						
18	84.4 ± 1^b^						
20	100 ± 0^a^						
*Bti*	100 ± 0^a^						
DMSO	0 ± 0						

**Bti:** *Bacillus thuringiensis israelensis*; **DMSO:** dimethyl sulfoxide; **LC**_50_ **:** lethal concentration to kill 50% of exposed larvae; **LC**_90_ **:** lethal concentration to kill 90% of exposed larvae; **LCL:** lower confidence limit; **UCL:** upper confidence limit; **χ** ^2^ **:** *Chi*-square not significant (*p*≥0.05); **d.f:** degrees of freedom; **SE:** standard error. *Letters in the same column indicate statistical difference (one-way ANOVA and Tukey’s test *p*<0.05).

The secondary metabolism of both plants and endophytic fungi produce 3-NPA and this may result from the interaction of both; therefore, it can be found in different parts of the host species, especially in the leaves, due to the higher occurrence of endophytic species in this organ^4^. Furthermore, 3-NPA of fungal origin is a precursor of other bioactive molecules of interest to the industrial sector and may present antifungal, antibacterial, antiviral, and insecticidal properties, among others^5^. 

At 20 ppm, 3-NPA caused 100% mortality in *Aedes aegypti* larvae after 24 hours of exposure. These results were compared with the activity of the bacterium Bti in the positive control, which also caused the death of all exposed larvae at 1 ppm. This bacterium exhibits high toxicity to the larvae of the genera *Aedes, Culex*, and *Anopheles*, and is one of the most suitable larvicides for the biological control of *Aedes aegypti* ^9^. Rotation of this bacterium with temephos is indicated to prevent resistance^10^. The World Health Organization^11^ recommends the use of Bti at concentrations ranging from 1 to 5 ppm, and the concentration used in this study was within the recommended range.

Temephos is an organophosphorus pesticide that is widely applied in domestic water reserves to control *Aedes aegypti* at a concentration of 1 ppm^11^. Its effects on human health are still uncertain; however, studies by Verdín-Betancourt et al.^12^ have shown that in chlorinated water, a stable product of temephos oxidation is a potent inhibitor of human acetylcholinesterase, suggesting high toxicological potential. Furthermore, organophosphates are not selective and affect non-target organisms, such as aquatic invertebrates, to which they can cause biological feeding and reproductive dysfunctions^13^. Therefore, it is important to search for other control products, and 3-NPA is considered a possible alternative. 

Few studies have investigated secondary metabolites of endophytic fungi that can be applied to the larvae of *Aedes aegypti*. To date, the study by Masi et al.^9^ is the only study that evaluates the larvicidal activity of 3-NPA*.* Their results indicated that 3-NPA isolated from the endophytic fungus *Diaporthe gulyae* causes 100% mortality in the first-stage larvae of *Aedes aegypti* at concentrations of 1000 ppm and 500 ppm, and 33.3% mortality at 250 ppm. These values are higher than those obtained in the present study where 3-NPA at 20 ppm caused 100% mortality of the third-stage larvae of *Aedes aegypti*. This disparity may be related to the different susceptibilities of the larval stages to 3-NPA, as observed in larvicidal assays with the compound fosinopril^14^. The results of this study showed that first-stage larvae were more resistant than second- and third-stage larvae, even after 72 hours of treatment with fosinopril. 

Masi et al*.* ^9^ did not evaluate lethal concentrations of 3-NPA; however, cytochalasin A and gliotoxin isolated from the fungus *Neosartorya pseudofischeri* (anamorph *Aspergillus thermomutatus*) were among the most effective substances evaluated against the first-stage larvae of *Aedes aegypti*, with LC_50_ of 85.4 ppm and 25.7 ppm, respectively in 24 hours. The results of the present study indicated that 3-NPA presented a lower lethal dose after 24 hours than the substances mentioned, as, at 15,172 ppm, it was able to kill 50% of the third-stage larvae of the species. 

Other substances from natural sources, such as plants, have also been evaluated against the larvae of *Aedes aegypti.* In a study by Nobsathian et al.^15^, lethal doses of cinnamic and 3,4-dimethoxybenzoic acids, isolated from *Maerua siamensis* (Capparidaceae) and applied in larvicidal assays on third-stage larvae of *Aedes aegypti*, were determined 48 hours after larval exposure. The acids presented LC_50_ of 22.79 and 72.54 ppm, and LC_90_ of 1581.11 and 2732.78 ppm, respectively^15^. In general, when considering the lethal doses of 3-NPA (LC_50_ = 15.172 ppm and LC_90_ = 18.178 ppm), this acid was more effective than the substances used in the aforementioned study. 

The results of this study suggest that 3-NPA can be used as a larval control agent for *Aedes aegypti*. Further studies should be conducted to investigate the differences in susceptibility of the larval stages of the species and establish the appropriate concentrations for these different stages. The results of the present study may be useful in the search for new larvicidal compounds as alternatives to temephos, and subsequently minimize larval resistance and environmental impacts, especially on aquatic organisms.
